# JBP at 50: “It is hard work, but deeply rewarding”

**DOI:** 10.36416/1806-3756/e20250442

**Published:** 2025-11-19

**Authors:** José Roberto Jardim

**Affiliations:** 1. Universidade Federal de São Paulo, Escola Paulista de Medicina, São Paulo (SP) Brasil.

Fifty years have passed since the first issue of the *Jornal de Pneumologia*, later renamed to *Jornal Brasileiro de Pneumologia* (JBP)*,* was published, and the first thought that comes to mind is: *“How quickly time passes!”* Out of curiosity, this reminds me of an article written by a Brazilian basic science researcher who discussed whether that statement was actually true. He explained that, of course, a day always has 24 hours, a week always has seven days, and a month always has about thirty days. The perception of time passing faster, he argued, is due to the accumulation of knowledge-as people grow older, their understanding of the world makes learning easier and faster. There is a clear parallel between that explanation and the history of the JBP. A scientific journal is a living organism, and the remarkable growth of the JBP over these 50 years is certainly grounded in the increasing expertise and experience of the Editors who have followed one another, of the support teams, and, of course, in the maturation of Brazilian researchers.

The JBP has a rich history, widely recounted through the testimonies of its various Editors-in-Chief when the Journal celebrated its 40th anniversary. Some of those testimonies were titled *“The JBP I Lived.”* At the time, for personal reasons, I was unable to write about my own experience with the *Jornal de Pneumologia*. I am deeply grateful to the current Editor-in-Chief of the JBP for this honorable invitation on the occasion of its 50th anniversary. I now feel compelled to share “*The JBP I Lived”* with our readers.

Let us go back in time 43 years. In 1982, I was invited to serve as the Editor-in-Chief of the *Jornal de Pneumologia,* and I officially took office at the closing session of the Brazilian Congress of the *Sociedade Brasileira de Pneumologia e Tisiologia* (SBPT), held in São Paulo, Brazil. The *Jornal de Pneumologia* was in its early years, and previous Editors had already made it a reality, keeping it alive and growing. The incoming SBPT board at that time established that the *Jornal de Pneumologia* should encourage Brazilian researchers to publish their studies in it, ensure regular publication, increase the number of articles published, and become the primary national source of information in respiratory medicine.

Several initiatives were then developed. During my two terms as Editor-in-Chief, we discussed the need to create a permanent board of peer reviewers. Until then, manuscripts were reviewed either by the Editor or by a pulmonologist invited on an ad hoc basis, without a formal peer review process or clear editorial guidelines. Even in those early days, the *Jornal de Pneumologia* was concerned about the misuse of common but incorrect terms, such as calling a disease a *pathology*-a word that properly refers to a medical specialty, not to a disease itself.

Not all authors submitting their manuscripts had prior research experience, and it was common for articles to lack a proper description of the Methods section or a well-structured Discussion. I recall one author who cited his own article as a reference in a foreign publication. I phoned him to explain that we could not publish an already published paper, to which he replied, “But it’s not the same article-it was published abroad *without* the photos!” To guide authors, the editorial team routinely sent reviewer comments accompanied by educational notes.

At that time, our journal was published only in Portuguese, with a few articles per issue-often including a series of educational review articles (for example, on physiology). It was printed using offset techniques, with photolithography (transparent film pages) and metal plates for the covers and content, allowing reprints when needed. It was an almost artisanal process, demanding great dedication from the graphic producer and printer. I must here express special thanks to Dr. Alexandre Moroz, a college contemporary from my days at *Escola Paulista de Medicina* and a fellow high hurdles competitor in academic contests, who served as a graphic producer from Volume 4 to Volume 10 of the Journal. Interestingly, after graduation, he also completed a fellowship in our Pulmonology Department, thus becoming a pulmonologist himself.

With approval from the SBPT board, Dr. Moroz and I developed a strategy to increase the Journal’s visibility: we mailed each new issue to all medical school and major hospital libraries in Brazil. Although this had financial costs, we believed it was worth it-and indeed, the number of manuscript submissions gradually began to increase.

During my tenure, the *Jornal de Pneumologia* applied for indexation in MEDLINE/PubMed for the first time. At that time, BIREME-the Regional Library of the Pan-American Health Organization-had a staff member directly linked to PubMed, who guided us through the application process. We sent a detailed letter describing SBPT, its objectives, achievements, and the Journal’s history, along with the four most recent volumes. The response, though polite, informed us that the Journal was yet to be eligible for indexation and advised us on the steps to be followed in the coming years. Those recommendations were gradually implemented, maintained, and improved upon by subsequent editorial teams. Finally, in 2006-after nearly 22 years-the JBP achieved PubMed indexation. It was a long gestation, but a necessary one, ensuring that the Journal met the rigorous standards of PubMed and that both the JBP Editors and SBPT leadership could sustain them.

Scientific journals are the open doors of professional societies, allowing them to reach the most distant parts of their countries and beyond. Each field of health science has its own journals, usually published by its respective professional societies, which aim to disseminate knowledge and best practices. In some countries, there is a strong integration among health professions, leading to joint publications and shared diagnostic and treatment guidelines. In Brazil, this integration is still far from ideal. Different societies often work in parallel toward similar goals, duplicating efforts and costs. I believe our Journal, the JBP, has the experience and credibility to serve as a unifying platform-one that promotes knowledge sharing across all respiratory health disciplines and fosters collaboration among medical specialties.

The next 50 years of the JBP have already begun. The current SBPT Board and Editor-in-Chief, Dr. Márcia Pizzichini, have set new challenges that build upon those faced and overcome by previous Editors-in-Chief. The Journal’s growing international recognition should further increase its impact factor; its leadership in Latin America will continue; the team of associate editors will expand; and publications grounded in rigorous scientific methodology will form the basis for new therapeutic recommendations, national guidelines, and public health strategies. These, in turn, will strengthen primary care practices and support Brazil’s Unified Health System.

Reflecting on my two terms as Editor-in-Chief, I once told a colleague-who had just been invited to take on the same role and asked if it was a lot of work-“Yes, it is hard work, but deeply rewarding.” I am certain that every Editor has felt the same way. Our Society (SBPT) deserves that work.


José Roberto Jardim Editor-in-Chief of the Jornal de Pneumologia, 1982-1984 and 1984-1986


